# Sugar Protectants Improve the Thermotolerance and Biocontrol Efficacy of the Biocontrol Yeast, *Candida oleophila*

**DOI:** 10.3389/fmicb.2019.00187

**Published:** 2019-02-08

**Authors:** Fangliang Zheng, Weiwei Zhang, Yuan Sui, Ruihan Ding, Wenfu Yi, Yuanyuan Hu, Hongsheng Liu, Chunyu Zhu

**Affiliations:** ^1^ School of Life Science, Liaoning University, Shenyang, China; ^2^ Chongqing Key Laboratory of Economic Plant Biotechnology, Institute of Special Plants, Chongqing University of Arts and Sciences, Yongchuan, China

**Keywords:** antioxidant enzyme, biocontrol yeast, gene expression, sugar protectants, thermotolerance

## Abstract

A variety of sugar compounds have been used as additives to protect various biocontrol yeasts from adverse environmental stresses. However, studies on maltose and lactose as sugar protectants are limited, and their protective effect is not clear. In the present study, exposure of the biocontrol yeast *Candida oleophila* cells to 45°C for 10 min, while immersed in either 5 or 10% (w/v) maltose or lactose, provided a significant protective effect. The addition of maltose and lactose significantly enhanced enzyme activity and gene expression of catalase, thioredoxin reductase, and glutathione reductase, relative to cells that have been immersed in sterile distilled water (controls) exposed to 45°C. In addition, *C. oleophila* cells suspended in maltose and lactose solutions also exhibited higher viability and ATP levels, relative to control cells. Notably, the biocontrol efficacy of *C. oleophila* against postharvest diseases of apple fruit was maintained after the yeast was exposed to the high temperature treatment while immersed in maltose and lactose solutions. These results demonstrate the potential of maltose and lactose as sugar protectants for biocontrol agent against heat stress.

## Introduction

Postharvest losses in apples from gray mold (*Botrytis cinerea*), blue mold (*Penicillium expansum*), and Alternaria rot (*Alternaria alternata*) are an ongoing problem (Romanazzi et al., 2016; Vilanova et al., 2017; Kim et al., 2018). Due to concerns about the health hazards of traditional postharvest fungicides, the use of postharvest biocontrol agents has been widely explored (Droby et al., 2009; Liu et al., 2013a; Spadaro and Droby, 2016). Biocontrol efficacy of the antagonists after application, however, is affected by various environmental conditions, including high temperature, salt stress, and oxidative stress (Ippolito and Nigro, 2000; Liu et al., 2011a, 2012; Sui et al., 2015; Cheng et al., 2016; Wang et al., 2018). In order to enhance the survival and performance of antagonistic yeasts exposed to adverse abiotic stresses, the addition of sugar protectants such as trehalose, fructose, lactose sucrose, galactose, and glucose (Abadias et al., 2001; Li and Tian, 2006; Melin et al., 2007; Sui and Liu, 2014) has been explored. The beneficial effect of specific sugar protectants, however, may be specific to each species of biocontrol agent. Therefore, there is a need to better understand the mechanism underlying the protective effect.

Sugars as protectants have been extensively studied. Maltose is a sugar protectant that is also used as a nutrient in yeast culture broth (Gientka et al., 2016). Lactose is a type of disaccharide that is present in mammalian milk (Chen and Gänzle, 2017). Maltose and lactose have a relatively low cost, which makes them ideal for use as a protectant additive in the formulation of yeast biocontrol products or the actual use of yeast biocontrol agents in commercial settings. Importantly, however, little research has been conducted on the use of these compounds as protectants of antagonistic yeast against adverse environmental conditions.

*Candida oleophila* is a yeast, whose postharvest biocontrol activity has been well-documented (Liu et al., 2012, 2013b; Wang et al., 2018). It can be applied to apple fruit before and after harvest for the management of postharvest pathogens such as *B. cinerea*, *P. expansum,* and *A. alternata*. The survival rate of *C. oleophila* is dramatically reduced, however, over 40°C (Liu et al., 2012). In response to high temperature stress, the level of reactive oxygen species (ROS) in yeast cells like *Saccharomyces cerevisiae* and *Pichia guilliermondii* rapidly accumulates, and oxidative damage of the mitochondria results in a significant decrease in intracellular ATP content, cell viability, and ultimately, yeast survival (Kim et al., 2013; Sui and Liu, 2014). The present study was conducted to determine: 1) the protective effect of maltose and lactose on *C. oleophila* exposed to lethal high temperature stress (45°C, 10 min); 2) the effect of these sugars on gene expression and enzyme activity of the antioxidant enzymes, catalase (CAT), thioredoxin reductase (TrxR), and glutathione reductase (GR); 3) changes in intracellular ATP levels in response to the sugars and temperature stress; 4) the biocontrol efficacy of *C. oleophila* against gray mold (*B. cinerea*), blue mold (*P. expansum*), and Alternaria rot (*A. alternata*) of apple after the yeast had been immersed in the sugar solution and exposed to a lethal, heat stress (45°C, 10 min).

## Materials and Methods

### Yeast Strain

The biocontrol yeast *C. oleophila* (strain I-182) was isolated from the surface of tomato fruit (Wilson et al., 1993). It was obtained from Dr. Michael Wisniewski at USDA-ARS-Appalachian Fruit Research Station, USA. About 100 ml of yeast peptone glucose (YPD) broth (10 g yeast extract, 20 g peptone, and 20 g glucose in 1,000 ml water) was prepared in a 300 ml Erlenmeyer flask and inoculated with *C. oleophila* to obtain an initial concentration of 1 × 10^5^ cells/ml. Yeast cultures were incubated at 25°C on a rotary shaker at 200 rpm for 16 h, at which time the cultures have reached the medium index phase, with an OD value of 0.7.

### Fungal Pathogens

The fungal pathogens, *B. cinerea* (CGMCC3.3790), *P. expansum* (CGMCC3.3703), and *A. alternata* (CGMCC3.15529), were obtained from China General Microbiological Culture Collection Center (CGMCC) and maintained on potato dextrose agar (PDA). To reactivate the culture and verify their pathogenicity, each pathogen was inoculated into wounded apple fruit and re-isolated onto PDA, once an infection was established. Spore suspensions of either *B. cinerea*, *P. expansum*, or *A. alternata* were obtained from 2-week-old PDA cultures grown at 25°C. Final spore concentrations were determined using a hemocytometer and adjusted to 10^4^ cells/ml with sterile distilled water.

### Fruit

Apple fruits were harvested at commercial maturity. Fruits without wounds or rot were selected based on the uniformity of size. The selected fruits were disinfected with 2% (v/v) sodium hypochlorite for 2 min, rinsed with tap water, and air-dried prior to their use in the biocontrol assays.

### Heat Treatment of Yeast Suspensions

Yeast cells were pelleted by centrifugation at 8,000 *g* for 3 min and washed three times with sterile distilled water to remove residual medium. Aliquots of the aqueous cell suspension were transferred to a conical flask (50 ml) containing 5 or 10% solutions (w/v) of either maltose or lactose to obtain a final concentration of 1 × 10^8^ cells/ml. A culture flask containing sterile water and 1 × 10^8^ yeast cells/ml was used as a control. All of the flasks were incubated in a 45°C water bath and manually shaken for 10 min. Immediately after the treatment, the treated yeast cells were then cooled by incubating the flasks in a 25°C water bath.

### Viability Assay

The sugar solution was removed from the treated samples by pelleting the cells. Subsequently, the cells were resuspended in sterile water and adjusted to a concentration of 1 × 10^8^ cells/ml. The cells were then dilution plated on YPDA (YPD, 20 g agar per well). Petri plates were incubated at 25°C for 12 h, and cell viability was expressed as a percentage of colony forming units (CFU) relative to untreated cells. There were three replicates in each treatment, and each experiment was repeated three times.

### RNA Isolation and Reverse Transcription–Quantitative PCR (RT-qPCR) Analysis of Gene Expression

Total RNA was extracted from each of the samples of treated yeast cells (water, maltose, or lactose at 5 and 10% (w/v) exposed to 45°C for 10 min). The total RNA extracts were treated with DNase and then purified using an EasyPure RNA Kit (TransGen Biotech, Beijing, China) according to the manufacturer’s instructions. Total RNA was then reverse transcribed into cDNA using TranScript One-Step gDNA Removal and cDNA Synthesis SuperMix (TransGen Biotech, Beijing, China). RT-qPCR analysis was conducted using GoTaq^®^ qPCR Master Mix kit (Promega, Madison, Wisconsin, USA). A qTOWER 2.2 (Analytik Jena AG, Germany) thermocycler was set to the following cycle: 94°C for 30 s, 94°C for 5 s, 58/59/60°C for 15 s, 72°C for 10 s, 40 cycles. The expression level of three target genes, *CAT*, *TrxR*, and *GR,* was analyzed using gene-specific primers (Table 1). The resulting data were normalized to a reference gene, *18S rRNA* (Liu et al., 2012). The 2^−▵▵CT^ method was used to calculate relative expression (Livak and Schmittgen, 2001). At the end of each PCR reaction, melting curve analyses of the amplification products were performed to ensure that unique products were amplified. PCR products were cloned and sequenced to verify their identity. The RT-qPCR analyses consisted of three independent biological replicates and three technical replicates per treatment, and the experiment was repeated three times.

**Table 1:** Gene-specific primers used in RT-qPCR analysis of gene expression.

Gene name	NCBI accession no.	Primer sequence	Annealing temp. (°C)	Product size (bp)
*CAT*	JN615130	F: AAGGGTTCAGGTGCTTACGG	59	140
		R: GCAGAACCGTTTTCACCACC		
*TrxR*	JN615133	F: TATCACCACCGATGCCGTTG	60	175
		R: ACATGCAGAGTCACCACCAC		
*GR*	JN615135	F: CATTGCTGCCGGACGTAGAT	58	123
		R: ATAGATCCGGCTTCTGGGTG		
*18S rRNA*	AB013534	F: ATTGGAGGGCAAGTCTGGTG	58/59/60	156
		R: AGAAGGAAAGGCTCGGTTGG		

### Assay of Enzyme Activity

Extracts from treated yeast cells for the enzyme activity assay of the antioxidant enzymes, CAT, GR, and TrxR, were prepared as described in the previous studies (Cheng et al., 2016; Wang et al., 2018). Untreated yeast cells (not immersed in a sugar solution) were used as a control. Cells were disrupted in liquid nitrogen and suspended in cold potassium phosphate buffer (0.1 M, pH 7.0). The cell homogenate was centrifuged at 10,000 *g* for 20 min at 4°C, and the resulting supernatant was used in each of the enzyme assays. The enzyme activity of CAT, TrxR, and GR was measured using commercial assay kits (Nanjing Institute of Bioengineering, Nanjing, China), and expressed as U per mg protein. Protein content was measured using a Bradford assay (Bradford, 1976) with bovine serum albumin as a standard. One unit of CAT activity was defined as the decomposition of 1 μM H_2_O_2_ per second in the reaction system. One unit of TrxR activity was defined as the deoxidation of 1 nM DTNB per min at 25°C. One unit of GR activity was defined as the amount necessary to decompose 1 mM of NADPH as a substrate per min. Assays of each treatment consisted of three independent biological replicates and the experiment was repeated three times.

### ATP Levels

The ATP assay was performed as described in a previous study (Li et al., 2010). ATP in *C. oleophila* cells (approximately 20 mg of fresh weight) was extracted with 50 μl of 2.5% trichloroacetic acid (TCA) for 3 h at 4°C. After centrifugation at 10,000 *g* for 15 min, 10 μl of the resulting supernatant was diluted with 115 μl of ATP-free H_2_O and 125 μl of ATP-free Tris-acetate buffer (40 mM, pH 8.0). ATP content was determined using a luciferin/luciferase kit (ENLITEN^®^ ATP Assay System, Promega, USA) according to the manufacturer instructions. Luminescence emission was determined using a multi-mode microplate reader. The assay of each treatment sample consisted of three, independent biological replicates, and the experiment was repeated three times.

### Biocontrol Assay

The biocontrol efficacy of *C. oleophila* against gray mold (*B. cinerea*), blue mold (*P. expansum*), and Alternaria rot (*A. alternata*) was evaluated. Three wounds (4 mm deep × 3 mm wide) were made on the equator of each fruit with a sterile nail. A 10 μl suspension (1 × 10^7^ cells/ml) of maltose-treated, lactose-treated, or non-sugar-treated *C. oleophila* yeast cells that have been exposed to 45°C for 10 min was pipetted into each wound. Sterile distilled water served as one control, while 10 μl suspension (1 × 10^7^ cells/ml) of fresh yeast cells not exposed to sugar and high temperature served as a second control. All the yeast suspensions applied into wounds were made in sterile distilled water. The yeast-treated fruits were separated into three groups. After the fruits were air-dried for 2 h, 10 μl of a *B. cinerea* suspension (1 × 10^4^ spores/ml) was inoculated into each wound of the first group, 10 μl of a *P. expansum* suspension (1 × 10^4^ spores/ml) was inoculated into each wound of the second group, and 10 μl of a *A. alternata* suspension (1 × 10^4^ spores/ml) was inoculated into each wound of the third group. Treated fruits were placed in a covered plastic food tray, and each tray was enclosed with a polyethylene bag and stored at 25°C. Disease incidence and lesion diameter on each apple were determined after 4 days. Incidence represented the percentage of infected wounds, while lesion diameter was measured only on those wounds that were infected. Each treatment contained three replicates with 10 fruits each, and the experiment was repeated three times.

### Data Analysis

All statistical analyses were performed using SPSS version 20.0 (SPSS Inc., Chicago, IL, USA). Data with a single variable (treatment) were analyzed using one-way ANOVA, and mean separations were performed using a Duncan’s multiple range test. Differences at *p* < 0.05 were considered as significant. Data presented in this article were pooled across three independent repeated experiments, as the interaction between treatment and experiment variables was not significant.

## Results

### Effect of Maltose and Lactose on Thermotolerance of *C. oleophila*

**Figure 1:**
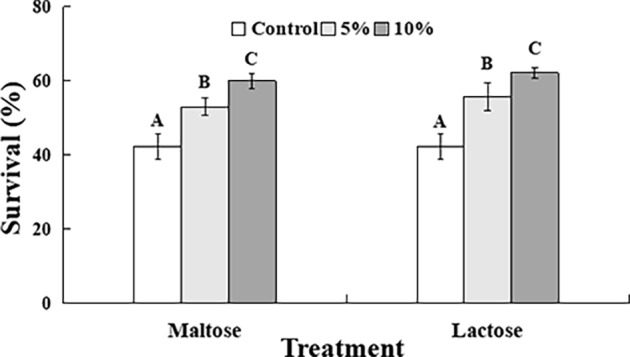
Percent survival of *C. oleophila* cells suspended in water (control), or 5 and 10% (w/v) maltose or lactose and exposed to 45°C for 10 min. Error bars represent standard deviations of the means. Data represent the mean ± standard deviation of three independent experiments, where each experiment consisted of three biological replicates (*n* = 9). Columns with different letters are significantly different according to a Duncan’s multiple range test at *p* < 0.05.

The survival rate of *C. oleophila* in the control samples after exposure to 45°C for 10 min was approximately 42% (Figure 1). The survival rate of yeast cells immersed in either the 5 or 10% maltose or lactose sugar solutions, however, was significantly increased. The survival rate in the 10% sugar treatments was approximately 60%, which was approximately 10% higher than it was in the 5% sugar treatments, where the survival rate was approximately 50%.

### Gene Expression in Response to the Heat Treatment

**Figure 2:**
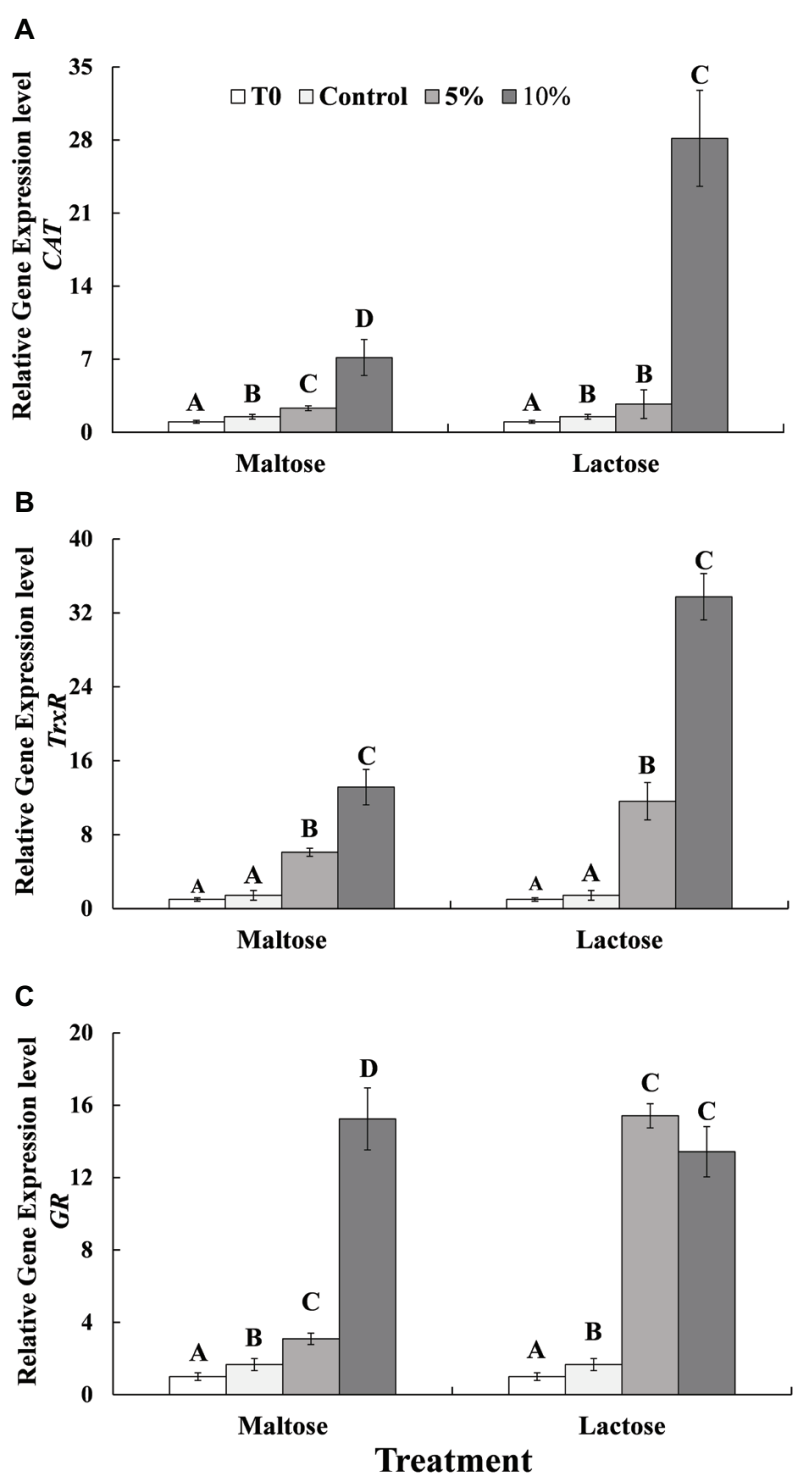
Relative expression of three antioxidant genes of *CAT* **(A)**, *TrxR* **(B)** and *GR* **(C)** in *C. oleophila* cells suspended in water (control), or 5 and 10% (w/v) maltose or lactose and exposed to 45°C for 10 min, and *C. oleophila* cells before heat treatment served as time 0 (T0). Data represent the mean ± standard deviation of three independent experiments, where each experiment consisted of three biological replicates (*n* = 9). Columns with different letters are significantly different according to a Duncan’s multiple range test at *p* < 0.05.

In general, exposure to heat stress significantly increased gene expression of *CAT and GR* (Control vs. Time 0, before heat treatment). The expression of *CAT* (Figure 2A) and *TrxR* (Figure 2B) exhibited in a similar fashion. Gene expression in the control group, 5% sugar treatment, and 10% sugar treatment increased in a stepwise manner, respectively; with the expression of *CAT* in the 10% lactose treatment being about 10 times greater than in the control and 5% lactose treatment cells. On the other hand, the expression in the 10% maltose treatment cells was significantly greater than in the control cells. The expression of *GR* (Figure 2C) also increased significantly in response to being immersed in the sugar protectants. *GR* expression in the 10% maltose treatment was approximately five times greater than in the 5% maltose treatment, while the expression in the 5% lactose treatment and 10% lactose treatment cells was basically similar and was significantly greater than in the control yeast cells.

### Antioxidant Enzyme Activity

**Figure 3:**
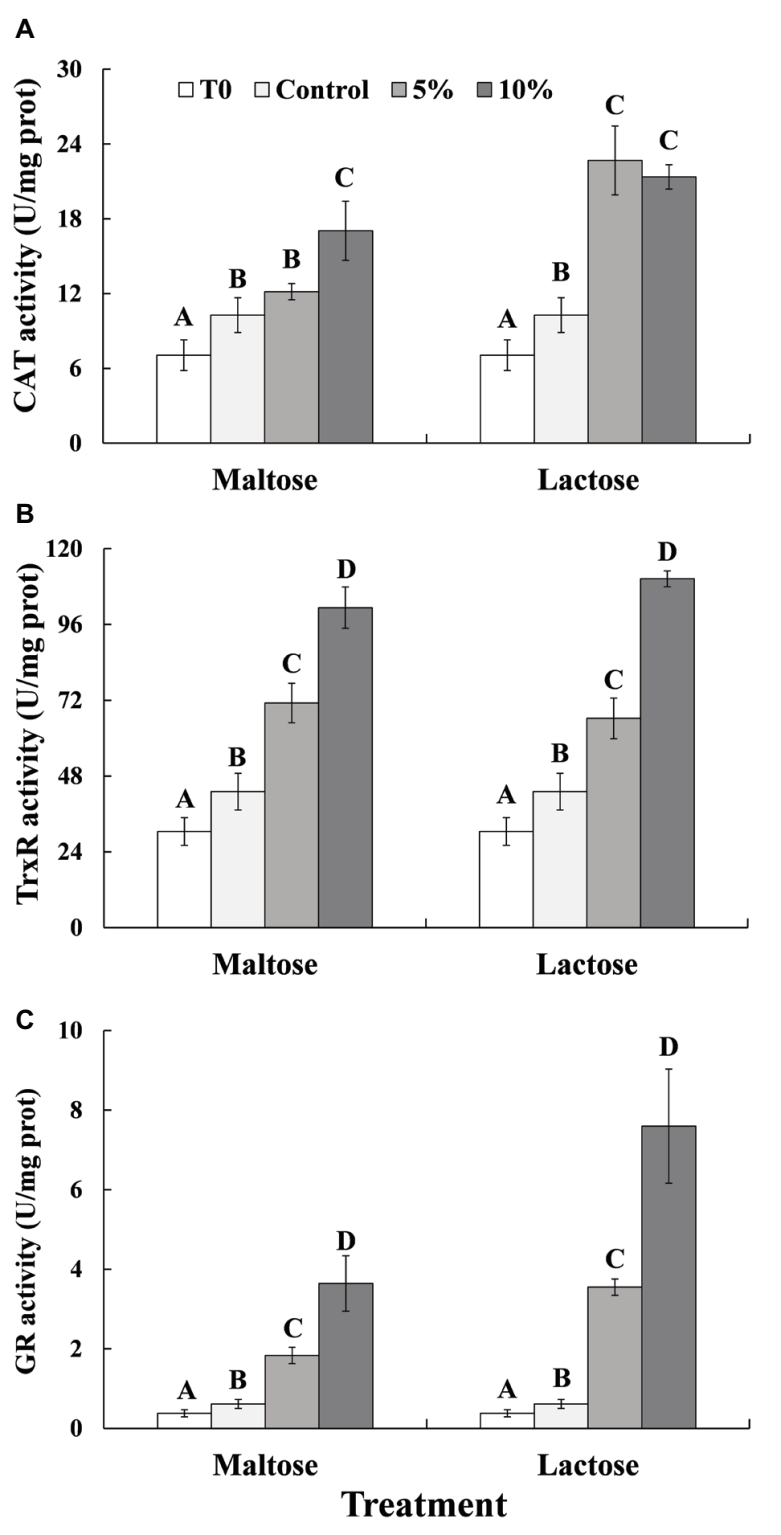
CAT **(A)**, TrxR **(B)**, and GR **(C)** activity in *C. oleophila* cells suspended in water (control), or 5 and 10% (w/v) maltose or lactose and exposed to 45°C for 10 min, and *C. oleophila* cells before heat treatment served as time 0 (T0). Data represent the mean ± standard deviation of three independent experiments, where each experiment consisted of three biological replicates (*n* = 9). Columns with different letters are significantly different according to a Duncan’s multiple range test at *p* < 0.05.

As indicated in Figure 3, exposure to heat stress significantly increased the activity of all the three enzymes, CAT, TrxR, and GR (Control vs. Time 0, before heat treatment). CAT enzyme activity in response to the heat treatment was significantly greater in the yeast cells that had been immersed in the sugar solutions than it was in the control cells that had been immersed in distilled water (Figure 3A). CAT enzyme activity was also significantly greater in the 10% maltose treatment than it was in the 5% maltose treatment. In contrast, CAT activity in the 5 and 10% lactose treatments was similar, though still twice the level than CAT activity in the control cells. TrxR (Figure 3B) and GR (Figure 3C) activities in cells of the 5% maltose treatment were approximately twice that of the control. TrxR and GR activities in the 10% maltose treatment were even higher than they were in the 5% maltose treatment and also significantly higher than the control treatment. In the 10% lactose treatment, TrxR activity was over double of the activity in the control. GR activity in 10% lactose treatment was approximately seven times greater than in the control treatment, about twice the level than 5% lactose treatment.

### ATP Levels of *C. oleophila* in Response to High Temperature

**Figure 4:**
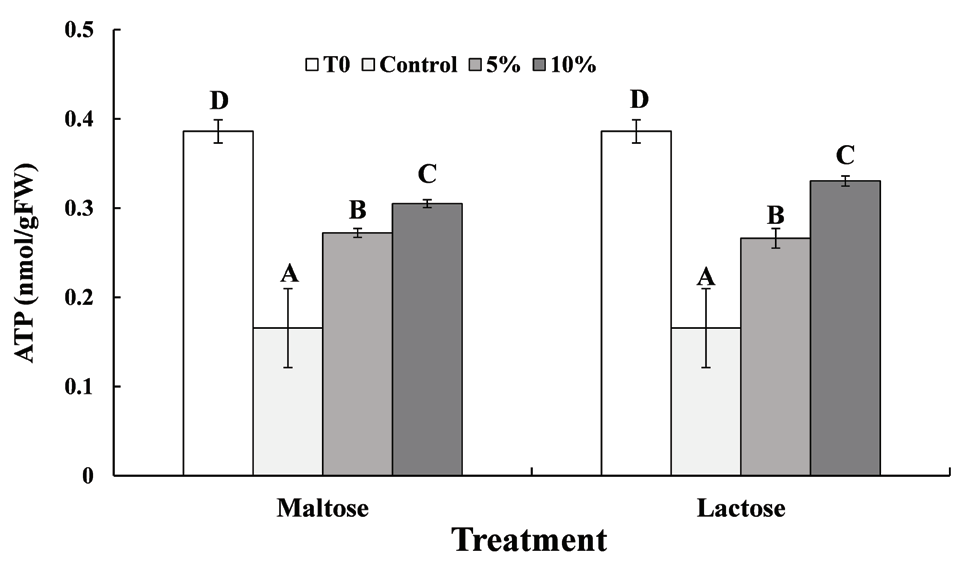
ATP levels measured in in *C. oleophila* cells suspended in water (control), or 5 and 10% (w/v) maltose or lactose and exposed to 45°C for 10 min, and *C. oleophila* cells before heat treatment served as time 0 (T0). Data represent the mean ± standard deviation of three independent experiments, where each experiment consisted of three biological replicates (*n* = 9). Columns with different letters are significantly different according to a Duncan’s multiple range test at *p* < 0.05.

ATP levels in *C. oleophila* cells immersed in the different sugar solutions before (Time 0) and after exposure to the high temperature treatment are presented in Figure 4. Heat stress at 45°C markedly decreased the ATP level in *C. oleophila* cells (Control vs. Time 0). The data from the maltose and lactose treatment were similar. ATP levels were three to four times higher than in the control samples. Additionally, the ATP content in the 10% sugar treatments was slightly higher than in the 5% sugar treatments. Collectively, the data indicate that the reduction in ATP levels induced by the high temperature treatment was inhibited by immersing the yeast cells in the sugar solutions, which acted as a protectant.

### Biocontrol Efficacy of *C. oleophila* Exposed to High Temperature

**Figure 5:**
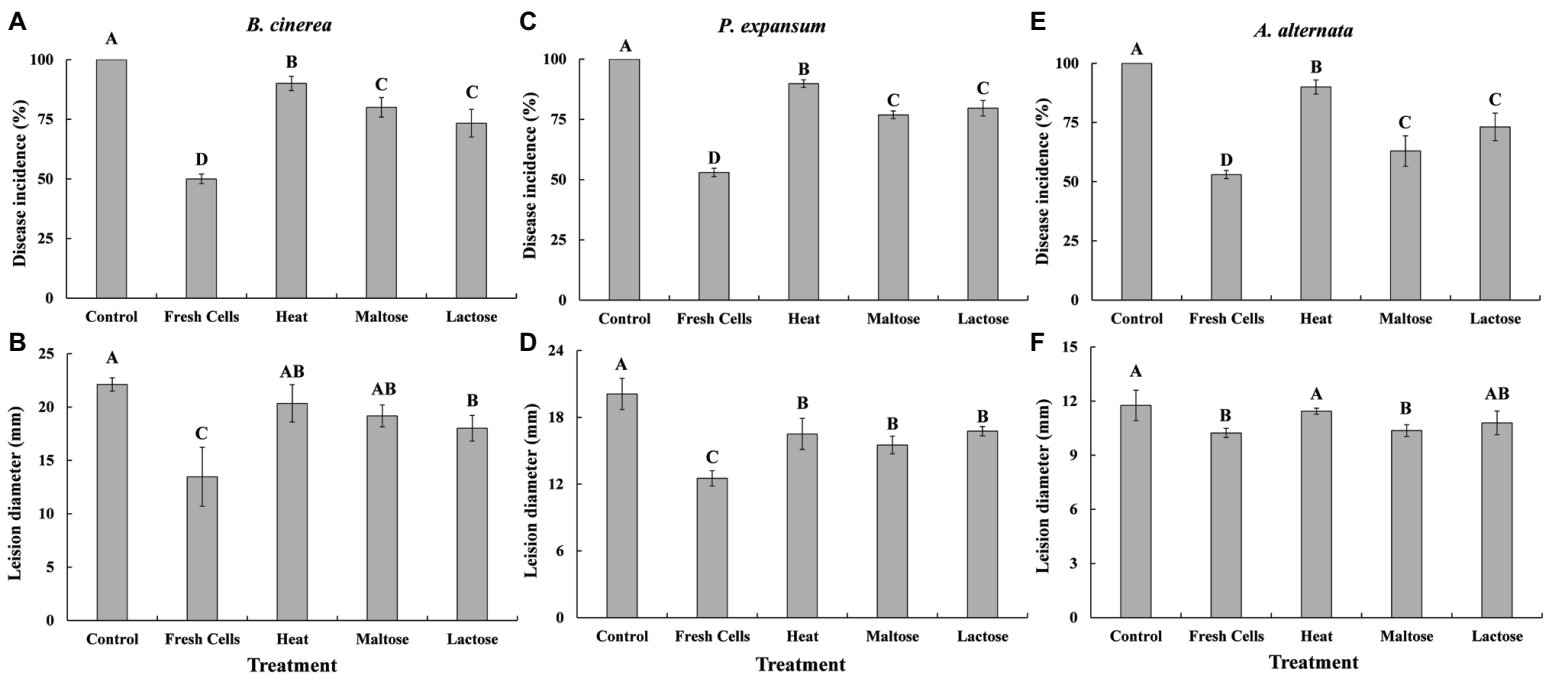
Biocontrol efficacy of *C. oleophila* against gray mold caused by *B. cinerea* **(A,B)**, blue mold caused by *P. expansum* **(C,D)**, and Alternaria rot caused by *A. alternata* **(E,F)** in apples stored at 25°C. Data represent the mean ± standard deviation of three independent experiments, where each experiment consisted of three biological replicates (*n* = 9). Columns with different letters are significantly different according to a Duncan’s multiple range test at *p* < 0.05.

Fresh cells that were not exposed to high temperatures effectively controlled gray mold caused by *B. cinerea* (Figures 5A,B), blue mold caused by *P. expansum* (Figures 5C,D), and Alternaria rot caused by *A. alternata* (Figures 5E,F) on apples stored at 25°C. Disease incidence in control apples that were not inoculated with yeast was 100%. In contrast, disease incidence in apples treated with yeast cells prior to inoculation with a pathogen was lower than the nonyeast controls. However, apples treated with yeast exposed to a high temperature stress while immersed in a sugar solution had a significantly lower disease incidence than apples treated with heat-treated yeast cells that had been immersed in water. Correspondingly, lesion diameters were generally smaller on fruits with fresh and sugar-treated cells than control and heat-treated yeast cells (non-sugar), though the difference was not as apparent as disease incidence.

## Discussion

In previous studies, the addition of sugar protectants maintained the viability and biocontrol efficacy of yeast antagonists (Melin et al., 2007; Sui and Liu, 2014; Niu et al., 2016). In the present study, it was clear that the elevated temperature was injurious to yeast cells. Maltose and lactose were effective in improving the tolerance of *C. oleophila* to high temperature stress (45°C, 10 min) (Figure 1). Temperature is an environmental parameter that can have a significant impact on the biocontrol efficacy of antagonistic yeasts used to manage postharvest diseases of fruit crops. Sugars are known to act as protectants against low temperature and high temperature stresses. In the present study, sugar protected the cells from an adverse stress (heat shock), and the adverse stress induced a resistance response that increased the general resiliency of the yeast cells to the conditions found in the wound sites of fruit (primarily osmotic and oxidative stress). In support of this premise, increased resistance to heat and oxidative stress was achieved by exposing biocontrol yeast or bacterial cells to a nonmetabolized, compatible solute, glycine betaine (Cañamás et al., 2007; Liu et al., 2011b; Sui et al., 2012; Zhang et al., 2017). Sugars can also be used as a protective agent in the preparation of liquid and dry formulations of biocontrol yeasts (Stephan et al., 2016). Direct interactions between proteins and the vitrification of sugars are considered to provide a protective mechanism (Sui and Liu, 2014).

Previous reports have demonstrated that heat stress also induces mitochondrial and oxidative damage in cells (Liu et al., 2011a, 2012; Sui and Liu, 2014). The present study demonstrated that ATP levels in *C. oleophila* cells decreased significantly by the heat treatment, but both maltose and lactose can significantly inhibit the reduction in ATP levels in *C. oleophila* cells that occur in response to high temperature exposure (Figure 4). Similar results were obtained by Sui and Liu (2014), who found that ATP of the biocontrol yeast, *Pichia guilliermondii*, decreased at 45°C, but the decrease trend was slowed down in glucose-treated cells. We speculate that mitochondrial damage caused by heat leads to reduced ATP levels. Mitochondria contain a high level of CAT, which can catalyze the decomposition of hydrogen peroxide into water (Martins and English, 2014). In the present study, CAT activity in maltose- and lactose-treated *C. oleophila* cells was significantly higher than in the control (Figure 3). GR in cell microsomes can catalyze the reduction of GSSG to GSH, reducing the oxidative levels in cells. TrxR can catalyze the reduction of DTNB by TAD and NADP^+^ by NADPH. The activity of both enzymes has the effect of reducing intracellular ROS levels (Gostimskaya and Grant, 2016). The present study demonstrated that both GR and TrxR in maltose-treated and lactose-treated *C. oleophila* cells exhibited higher activity than yeast cells in the control group. We suggest that these enzymes play a role in reducing ROS levels induced in the cells in response to heat stress and thus enhance survival. The expression of the corresponding genes to these enzymes was also higher in the sugar-treated cells than control cells (Figure 2) and corresponded to increase in enzyme activity (Figure 3).

The use of yeast antagonists with high viability for postharvest biocontrol is an advantage as competitive for nutrients, and space is one of the basic modes of action in postharvest biological control systems (Wilson and Wisniewski, 1989; Droby et al., 2016). In general, maltose- and lactose-treated *C. oleophila* cells exposed to a heat stress had lower biocontrol efficacy as fresh cells against gray mold, blue mold, and Alternaria rot, but higher efficacy than non-sugar-treated yeast cells exposed to a heat stress (Figure 5). Suspensions of yeasts were prepared to get the same cell numbers, but the percentage of living cells was not the same. Different numbers of living yeasts were applied in each treatment. This could be the cause of the lower biocontrol activity of heat-treated *C. oleophila*, since living cells are needed to exert the control of pathogens. The disease symptom on fruit host was determined by three bio-factors (pathogen-biocontrol agent-host fruit) of postharvest biocontrol system, and environmental factors (Liu et al., 2013a). In the present study, the differences in the protective effects of *C. oleophila* against these three different fungal species may be related to specific pathogenicity of each pathogen.

The collective data suggest that maltose and lactose can effectively reduce the oxidative damage that occurs in *C. oleophila* cells exposed to a lethal high temperature, and as a result, helps to maintain a high viability, survival, and biocontrol efficacy of the yeast. Collectively, the results of the present study demonstrate the ability of maltose and lactose to protect *C. oleophila* yeast cells against high temperature stress. However, further study on the molecular mechanism involved is needed.

## Author Contributions

YS and CZ conceived and designed the experiments. FZ, WZ, RD, and WY performed the experiments. FZ, WZ, YH, and HL analyzed the data. YS and CZ drafted the manuscript. All authors read and approved the final manuscript.

### Conflict of Interest Statement

The authors declare that the research was conducted in the absence of any commercial or financial relationships that could be construed as a potential conflict of interest.
